# An epidemiological assessment of the distribution and sociodemographic burden of chronic diseases: a focus on hypertension, diabetes, and cardiovascular conditions

**DOI:** 10.3389/fepid.2026.1715893

**Published:** 2026-05-22

**Authors:** Chidimma Evangeline Oliobi, Dawit Getnet Ayele, Knowledge Chinhamu, Temesgen Zewotir, Retius Chifurira

**Affiliations:** 1 School of Mathematics, Statistics and Computer Science, College of Agriculture, Engineering and Science, University of KwaZulu-Natal, Durban, South Africa; 2 Center for Policy, Planning, and Evaluation (CPPE), District of Columbia Department of Health, Washington, DC, United States

**Keywords:** cardiovascular disease, chronic disease prevalence, diabetes, epidemiologic analysis, hypertension, NHIS

## Abstract

**Background:**

Chronic diseases remain a major contributor to morbidity and mortality worldwide. Understanding the sociodemographic and behavioral factors associated with these conditions, as well as potential effect modification across population subgroups, is essential for developing targeted prevention strategies.

**Methods:**

We conducted a cross-sectional analysis of nationally representative survey data to examine the associations between sociodemographic and behavioral factors and hypertension, diabetes, and cardiovascular disease (CVD). Survey-weighted multivariable logistic regression models were fitted for each outcome. Restricted cubic splines were used to assess the non-linearity of age. An age × gender interaction term was evaluated, and predicted probabilities were estimated to visualize effect modification. The issue of missing data was addressed using multiple imputation (five imputations). Model discrimination and calibration were assessed using the area under the receiver operating characteristic curve (AUC) and calibration plots.

**Results:**

Advancing age was strongly associated with higher odds of all three conditions. Significant age × gender interactions were observed for diabetes and CVD, indicating steeper age-related increases in risk among males compared with females. In contrast, age-related increases in hypertension were similar across genders. Sociodemographic and behavioral factors such as income and BMI were independently associated with cardiometabolic outcomes. Model discrimination was good for hypertension (AUC = 0.80), diabetes (AUC = 0.77), and CVD (AUC = 0.83), with adequate calibration across risk deciles. Sensitivity analyses yielded consistent findings.

**Conclusions:**

Cardiometabolic risk is strongly associated with age and sociodemographic and behavioral factors, with important gender differences in age-related trajectories for diabetes and CVD. These findings underscore the importance of accounting for interaction effects in epidemiologic analyses and support age- and gender-tailored prevention strategies at the population level.

## 1 Introduction

Chronic diseases such as hypertension, diabetes, and cardiovascular disease (CVD) persist as major public health challenges worldwide, with unequal distribution across demographic and geographic groups. These health conditions are leading contributors to morbidity and mortality, resulting in diminished quality of life, elevated healthcare expenses, and substantial socioeconomic burden. Notwithstanding improvements in medical care and public health awareness, their prevalence continues to be disturbingly elevated, especially among certain communities (1, 2). Hypertension is defined as persistently elevated blood pressure (SBP ≥ 140 mmHg and/or DBP ≥ 90 mmHg) (3). This disease, also known as the “silent killer,” affects millions of people globally and is a significant risk factor for both diabetes and CVD (4, 5). Diabetes is a metabolic disease characterized by blood glucose levels that are higher than normal, resulting from deficiencies in either insulin production or insulin function (6). This disease, having systemic implications, has become more common, particularly in older people and those who lead sedentary lives (7). CVD, which includes a variety of heart and vascular disorders, remains the leading cause of mortality worldwide, with well-documented links to genetic, lifestyle, and environmental factors (8, 9). According to the American Heart Association's (10) statistical report, in 2022 (the most recent year for which final data are available), the overall number of cardiovascular disease (CVD)-related deaths in the United States was 941,652, an increase of more than 10,000 from the 931,578 CVD deaths in 2021. The most prevalent cause of human death worldwide is non-communicable disease (NCD), which leads to more than 41 million deaths annually, or 74% of all mortality (11, 12). NCD has resulted in the “double burden” of disease, wherein health systems are struggling with both high incidence of infectious diseases and rising incidence of non-communicable diseases such as hypertension, diabetes, and cardiovascular disease (13–16). This dual burden is especially evident in sub-Saharan Africa, where rapid urbanization and lifestyle transitions are accelerating the prevalence of cardiometabolic risk factors (17, 18). Population-based data are essential for identifying at-risk groups and guiding targeted prevention strategies, resource allocation, and health system planning (19). Digital health and mobile-based interventions have also been proposed as scalable strategies for improving chronic disease prevention and management in resource-constrained settings (20). Prior studies have highlighted the impact of both modifiable risk variables such as income, nutrition, and physical activity and non-modifiable ones such as age and gender (21, 22). These patterns are further shaped by contextual factors such as neighborhood deprivation and structural inequalities in access to healthcare (23). However, more research is needed to fully understand the intersectionality of these factors, especially how they interact with regional and racial differences. While the Southern “Stroke Belt” has historically demonstrated elevated cardiovascular mortality, geographic inequality in chronic disease extends beyond regional boundaries (24). Urban–rural gradients, healthcare access, and insurance coverage are further associated with variation in disease detection and management. Although NHIS data do not allow a fine-grained county-level analysis, regional comparisons provide insight into macro-level spatial heterogeneity.

In this study, disparities are operationalized as adjusted odds ratios across sociodemographic strata derived from multivariable logistic regression models. The analysis does not attempt causal decomposition but instead estimates relative differences in disease probability across demographic groups.

Although extensive data exist on sociodemographic factors associated with chronic disease in the United States, as well as epidemiologic trends in NCDs, most studies evaluate these conditions independently, often using heterogeneous modeling approaches or focusing on specific subpopulations. As a result, comparative evidence regarding whether sociodemographic gradients operate similarly or differently across these major chronic diseases remains limited. Without harmonized modeling approaches applied across conditions, it is difficult to determine whether observed inequalities reflect shared patterns of social stratification or disease-specific vulnerability profiles.

Although prior studies have provided valuable insights into individual chronic conditions, they have rarely examined hypertension, diabetes, and cardiovascular disease within a unified analytic framework that enables direct comparison of sociodemographic gradients across outcomes. In addition, more existing work relies on descriptive or condition-specific modeling approaches, limiting the ability to assess whether observed inequalities reflect consistent structural patterns or disease-specific dynamics. To address these limitations, the present study adopts a comparative, survey-weighted analytic framework that applies harmonized multivariable models across multiple chronic conditions. By incorporating interaction analyses, multiple imputation, and model performance evaluation, this study extends beyond conventional prevalence estimation to provide a more integrated and methodologically rigorous assessment of chronic disease burden.

This study is grounded in a perspective of social determinants of health, which conceptualizes age, sex, race/ethnicity, socioeconomic status, and geographic context as patterned dimensions of social organization that are associated with chronic disease prevalence. These dimensions are commonly interpreted in the literature as reflecting broader structural and contextual conditions, including differences in healthcare access, environmental exposures, resource availability, and life course experiences. However, in this cross-sectional analysis, they are treated as measured characteristics associated with disease prevalence rather than causal mechanisms. Within this framework, sociodemographic variables are not treated as merely descriptive characteristics but as structured gradients that may manifest differently across health outcomes. However, in cross-sectional survey analyses, they are examined as associations rather than causal pathways.

Accordingly, this study conducts a comparative epidemiological assessment of hypertension, diabetes, and CVD among US adults aged 18 years and older using harmonized, survey-weighted multivariable logistic regression models. Specifically, we aim to: (1) estimate the prevalence of each condition across key demographic strata; (2) examine adjusted associations between sociodemographic characteristics and each outcome using harmonized regression models; (3) evaluate heterogeneity in these associations through interaction analyses; and (4) assess and compare model performance across conditions. By applying consistent analytic specifications across outcomes, this study provides a systematic comparison of sociodemographic gradients in chronic disease.

Understanding whether chronic disease inequalities are structurally consistent or vary by disease has important implications for public health policy and intervention design. Identifying which demographic population groups experience the greatest burden across multiple conditions can inform more efficient targeting of prevention strategies, healthcare resources, and policy interventions. Through a unified and methodologically rigorous analytic framework, this study contributes comparative evidence to support data-driven and equity-oriented chronic disease prevention efforts. The government and health planners can plan for finance, infrastructure, and manpower for populations with a higher observed burden using reliable epidemiological data (25). The results of this study will be an auspicious addition to American research on the epidemiology of chronic disease, which, while increasing, remains limited and fragmented (13).

## 2 Methods

### 2.1 Study design and data source

This research utilizes data from the National Health Interview Survey (NHIS), a large-scale national household survey employed to monitor the health of non-institutionalized civilians. The NHIS is conducted annually by the National Center for Health Statistics, which is a component of the US Centers for Disease Control and Prevention. The data analyzed emanated from the harmonized NHIS data provided by the Integrated Public Use Microdata Series (IPUMS Health Surveys). The NHIS employs a multistage, stratified, cluster sampling design to produce population-level health estimates. Pooling multiple survey years (2020–2023) increased statistical precision and enabled stable subgroup estimates. The analysis was restricted to adults above 18 years with available information on study outcomes and key covariates. Three self-reported physician-diagnosed chronic conditions were examined as binary outcomes (i.e., hypertension, diabetes, and cardiovascular disease). Each outcome was coded as 1 (yes) or 0 (no) based on participants’ responses to standardized NHIS questions regarding prior diagnosis by a healthcare professional. Because the NHIS relies on self-report, outcomes reflect diagnosed disease rather than undetected or subclinical conditions. For the covariates, sociodemographic variables were included (i.e., age, sex, race/ethnicity, income, and region), as well as behavioral and clinical covariates [i.e., Body mass index (BMI), smoking status, insurance status]. The covariates were selected *a priori* based on established associations with chronic disease risk (26–30) and availability across survey years. The size of the dataset was 147,430. Missing data among variables included in the final analytic models were minimal (<1% for all covariates and outcomes). Multiple imputation by chained equations (MICE) was implemented as part of a broader analytic workflow using an extended dataset that included additional behavioral and healthcare access variables relevant to subsequent analyses. Although imputation was not strictly necessary for the present analysis, its inclusion ensured consistency across analytic components and improved statistical efficiency. A sensitivity analysis comparing imputed and complete-case models demonstrated consistent estimates, indicating that missing data did not materially influence the findings. Sampling weights were applied where necessary to yield population-level inference. Weighted estimates were representative of the US adult population during the pooled survey years. Descriptive statistics, regression models, and predicted probabilities were calculated using survey-weighted methods to ensure unbiased inference about the population.

### 2.2 Data analysis

#### 2.2.1 Descriptive analysis

Weighted prevalence estimates were calculated for each outcome and across sociodemographic strata. Differences in crude prevalence were interpreted as population-level distributions and not as adjusted associations.

#### 2.2.2 Multivariable logistic regression

Separate multivariable logistic regression models were fitted for hypertension, diabetes, and CVD using survey-weighted generalized linear models with a quasibinomial family to account for potential overdispersion. All models included age, sex, race/ethnicity, income quintile, region, BMI, and smoking status. Adjusted odds ratios (aORs) and 95% confidence intervals (CIs) were estimated. Because this study is cross-sectional, estimated associations reflect conditional relationships between covariates and diagnosed disease and should not be interpreted as causal effects.

The logistic regression model expresses the probability of a binary outcome as a function of one or more predictor variables (31). As shown in Equation 1, the log-odds of the outcome are modeled as a linear combination of the predictors:

$$\mathrm{Logit}(P)=\log\left(\frac{\;p}{1-p}\right)=\beta_{0}+\beta_{0}X_{1}+\beta_{0}X_{2}+\cdots +\beta_{k}X_{k}$$ (1)

where pistheprobabilityoftheeventoccurring, p/(1−p)representstheoddsoftheevent, log⁡(p/(1−p))representsthelogoftheodds, $\beta_{0}\;\mathrm{is}\;\;\mathrm{the}\;\;\mathrm{intercept}$, $\beta_{1},\beta_{2},\ldots ,\beta_{K}\;\mathrm{represent}\;\mathrm{the}\;\mathrm{coefficients}\;\mathrm{of}\;\mathrm{predictor}\;\mathrm{variables}$, and $X_{1},X_{2},\ldots ,X_{k}\;\;\mathrm{represent}\;\mathrm{the}\;\mathrm{predictor}\;\mathrm{variables}$.

#### 2.2.3 Assessment of non-linearity

To evaluate potential non-linear associations between age and each outcome, restricted cubic splines (natural splines with four degrees of freedom) were incorporated into regression models. The model fit was compared with linear specifications, and spline functions were retained where appropriate.

#### 2.2.4 Interaction analysis

To assess heterogeneity in associations, interaction terms were tested between age and sex and between race/ethnicity and income quintile. Interaction effects were evaluated using Wald tests. Statistically significant interactions were interpreted as evidence of effect modification of associations rather than causal structural mechanisms.

#### 2.2.5 Model discrimination and calibration

Model discrimination was evaluated using receiver operating characteristic (ROC) curves and area under the curve (AUC) statistics based on predicted probabilities from survey-weighted models. AUC values between 0.70 and 0.80 were interpreted as moderate discrimination. Calibration was assessed using the Hosmer–Lemeshow goodness-of-fit test and a graphical comparison of observed and predicted probabilities across deciles of risk. Given the sample size, calibration statistics were interpreted cautiously. Models were not developed as clinical prediction tools; discrimination metrics are reported to contextualize model performance.

#### 2.2.6 Predicted probabilities

Predicted probabilities were generated from fitted models to illustrate age gradients and interaction patterns. Marginal predicted probabilities were derived while holding covariates at observed values.

#### 2.2.7 Missing data and multiple imputation

Missing covariate data were addressed using MICE under a missing-at-random assumption. Five imputed datasets were generated using predictive mean matching for continuous variables and logistic regression for categorical variables. Regression models were fitted within each imputed dataset and pooled using Rubin's rules. The results from imputed analyses were compared with complete-case analyses as a sensitivity assessment.

#### 2.2.8 Sensitivity analysis

To assess the robustness of the findings, sensitivity analyses were performed by comparing complete-case regression models with multiple imputation models (pooled across five imputations). Also, a comparison of linear vs. spline age modeling was done. Consistency in direction and magnitude of associations across specifications was interpreted as evidence of analytic robustness.

### 2.3 Statistical software

All analyses were conducted in R (R Foundation for Statistical Computing) using the survey, mice, pROC, rms, and tidyverse packages. Statistical significance was defined as a two-sided *p*-value <0.05.

## 3 Results

### 3.1 Sample characteristics

The weighted analytic sample represented approximately 918.9 million US adults. The overall rates of prevalence of hypertension were 31.2% (95% CI: 30.8%–31.7%), diabetes 9.2% (95% CI: 9.0%–9.5%), and CVD 4.8% (95% CI: 4.7%–5.0%). Table 1 presents weighted characteristics of the study population stratified by hypertension status.

**Table 1:** Weighted characteristics of the study population by hypertension status.

Characteristic	Hypertension = 0 (no)	Hypertension = 1 (yes)
	*N* = 632,002,128	*N* = 286,886,932
Age (standardized), median (IQR)	−0.72 (−1.32, 0.10)	0.48 (−0.12, 1.03)
Gender, *n* (%)		
Female	301,859,180 (48%)	147,012,831 (51%)
Male	330,142,947 (52%)	139,874,102 (49%)
Race/ethnicity, *n* (%)		
American Indian	5,978,837 (0.9%)	2,753,064 (1.0%)
Asian	42,450,560 (6.7%)	13,329,095 (4.6%)
Black/African American	66,205,625 (10%)	42,773,308 (15%)
White	456,348,781 (72%)	208,941,805 (73%)
Other	15,473,110 (2.4%)	5,618,455 (2.0%)
Unknown/refused	45,545,215 (7.2%)	13,471,205 (4.7%)
Income, *n* (%)		
Low	75,297,711 (12%)	39,252,353 (14%)
Middle	477,103,131 (75%)	218,379,394 (76%)
High	79,601,286 (13%)	29,255,185 (10%)
Region, *n* (%)		
Midwest	131,464,123 (21%)	59,413,914 (21%)
Northeast	111,811,426 (18%)	49,147,928 (17%)
South	229,924,461 (36%)	119,314,430 (42%)
West	158,802,118 (25%)	59,010,661 (21%)
BMI category, *n* (%)		
Underweight	7,389,695 (1.2%)	1,437,026 (0.5%)
Normal weight	236,584,607 (37%)	57,998,466 (20%)
Overweight	223,205,786 (35%)	103,417,938 (36%)
Obese	164,822,040 (26%)	124,033,502 (43%)
Smoking (standardized), median (IQR)	−0.09 (−0.09, −0.09)	−0.09 (−0.09, −0.09)

Values represent weighted counts and percentages unless otherwise specified. Continuous variables are presented as median (IQR).

Participants with hypertension were substantially older, with a median standardized age of 0.48 (IQR −0.12–1.03) compared with those without the outcome, −0.72 (IQR −1.32–0.10). The distribution of gender between the groups is broadly comparable; however, females had a slightly higher proportion of people with hypertension (51% vs. 48%). Age-stratified analyses indicated that females were overrepresented in older age groups, where cardiometabolic disease prevalence is highest. Racial/ethnic differences were evident. Black/African American participants represented 15% of those with the outcome compared with 10% among those without, whereas Asian participants comprised a small proportion of affected individuals (4.6% vs. 6.7%). Income gradients were observed, with individuals in the lowest income tertile representing a greater proportion of those with hypertension (14% vs. 12%), while those in the highest tertile were underrepresented (10% vs. 13%). Geographic distribution differed modestly. Participants residing in the South accounted for 42% of those with the outcome compared with 36% among those without. Marked differences were observed across BMI categories. Obesity was substantially more prevalent among those with hypertension (43%) compared with those without (26%), whereas normal weight was less common among affected individuals (20% vs. 37%). Smoking exposure distributions were similar across groups.

### 3.2 Crude prevalence patterns

The weighted crude prevalence of hypertension, diabetes, and CVD was examined across sociodemographic and behavioral groups (Table 2).

**Table 2:** Weighted crude prevalence (%) of hypertension, diabetes, and cardiovascular disease across sociodemographic groups.

Variable	Category	Hypertension % (SE)	Diabetes % (SE)	CVD % (SE)
Age group (years)	18–39	9.3 (0.22)	1.6 (0.08)	0.4 (0.04)
	40–59	30.7 (0.33)	8.9 (0.20)	2.6 (0.11)
	60–79	56.3 (0.33)	18.6 (0.28)	10.9 (0.20)
	80+	67.6 (0.62)	19.2 (0.56)	21.5 (0.57)
Gender	Male	32.8 (0.29)	9.7 (0.17)	6.2 (0.13)
	Female	29.8 (0.28)	8.7 (0.16)	3.5 (0.09)
Race/ethnicity	American Indian	31.5 (1.85)	13.7 (1.57)	4.6 (1.34)
	Asian only	23.9 (0.75)	10.0 (0.48)	2.9 (0.27)
	African American	39.2 (0.63)	12.4 (0.39)	4.2 (0.23)
	White only	31.4 (0.25)	8.5 (0.13)	5.3 (0.10)
BMI category	1 (Normal)	16.3 (0.13)	2.9 (0.07)	2.6 (0.05)
	2 (Overweight)	19.7 (0.14)	4.5 (0.14)	3.8 (0.12)
	3 (Obese I/II)	31.7 (0.18)	8.7 (0.18)	5.1 (0.13)
	4 (Obese III)	42.9 (0.34)	14.7 (0.25)	5.7 (0.14)
Income	Low	34.3 (0.59)	12.4 (0.36)	6.0 (0.23)
	Middle	31.4 (0.24)	9.2 (0.13)	4.8 (0.10)
	High	26.9 (0.47)	5.9 (0.23)	3.7 (0.17)
Region	Midwest	31.1 (0.49)	8.9 (0.27)	5.0 (0.18)
	Northeast	30.5 (0.51)	8.7 (0.28)	5.0 (0.20)
	South	34.2 (0.36)	10.3 (0.21)	5.4 (0.14)
	West	34.2 (0.36)	8.2 (0.26)	3.6 (0.15)

Values are weighted prevalence (%) with standard errors in parentheses.

Overall, the rates of weighted prevalence in the US population were 31.2% for hypertension, 9.2% for diabetes, and 4.8% for CVD. The results showed that the hypertension prevalence rate rose from 9.3% among adults aged 18–39 years to 67.6% among those aged 80 years and older. The diabetes prevalence rate increased from 1.6% to 19.2% across the same age groups, while cardiovascular disease showed the steepest gradient, rising from 0.4% in the youngest group to 21.5% in the oldest group. These indicate a strong age gradient in the crude distribution of all three chronic conditions. With regard to gender, hypertension was slightly more prevalent among males than females, whereas diabetes prevalence showed a modestly higher burden among females. The prevalence of CVD was similar in both sexes. Racial/ethnic patterns showed that Black American adults had the highest prevalence of hypertension (39.25), followed by American Indians (31.5%) and Whites (31.4%). American Indians had the greatest prevalence of diabetes (3.7%), whereas CVD prevalence showed a similar ranking, with Black Americans slightly higher than other groups. Furthermore, the prevalence of hypertension increased with BMI increase, rising from 16.3% among normal weight persons to 66.1% among obese class III individuals. The BMI-associated gradients for diabetes and CVD were comparable. Adults with lower incomes had a slightly higher prevalence of hypertension and diabetes, while regional variation was evident, with the South showing the highest burden for all three conditions. These unadjusted prevalence patterns highlight substantial sociodemographic differences, which are further evaluated in multivariable models in Section 3.3.

**Figure 1:**
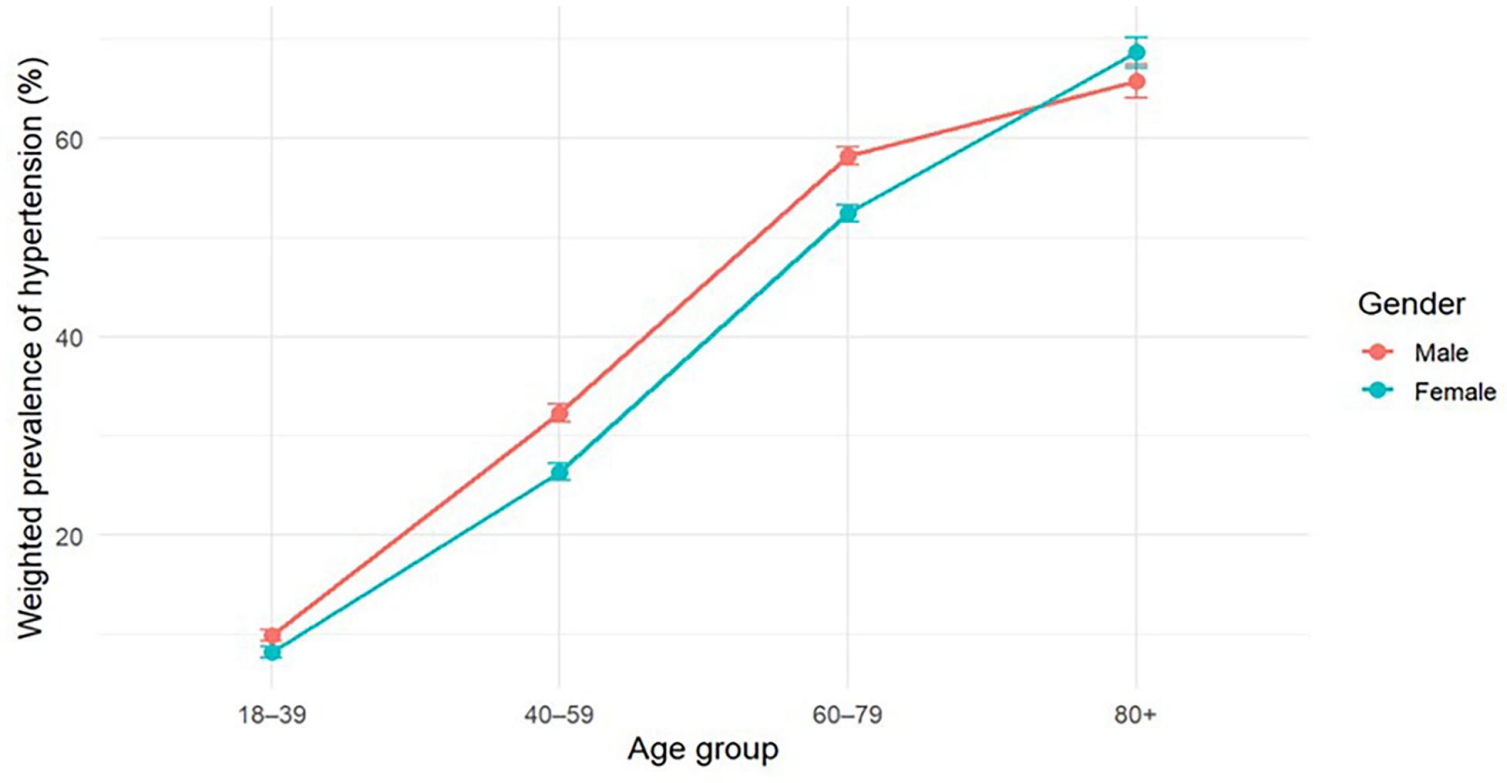
Age-stratified weighted prevalence of hypertension by gender.

Age-stratified prevalence patterns by gender are illustrated in Figure 1. Hypertension prevalence increased markedly across age groups for both genders. Males exhibited higher prevalence in most age categories, whereas females exceeded males only in the oldest age group (80+), indicating that the higher crude prevalence observed among women reflects differences in age distribution rather than uniformly higher gender-specific prevalence.

Error bars represent 95% confidence intervals. Corresponding numerical estimates are provided in Supplementary Table S1.

### 3.3 Multivariable associations

Restricted cubic spline analyses did not demonstrate meaningful departures from linearity for age across outcomes. The model fit did not show any improvement relative to linear specifications; therefore, age was retained as a linear term in all final models. Table 3 presents the results of the multivariable analyses performed.

**Table 3:** Adjusted associations between sociodemographic factors and chronic diseases (odds ratios [95% CI]).

Variable	Category	Hypertension OR (95% CI)	Diabetes OR (95% CI)	CVD OR (95% CI)
Main effects				
Age (per SD increase)	–	3.30 (3.23–3.37)	2.76 (2.68–2.85)	4.29 (4.08–4.52)
Gender	Male	Ref	Ref	Ref
	Female	0.80 (0.77–0.83)	0.82 (0.78–0.87)	0.46 (0.43–0.49)
Race/ethnicity	White	Ref	Ref	Ref
	Asian	0.86 (0.70–1.05)	1.04 (0.78–1.37)	0.68 (0.37–1.24)
	African American	1.22 (1.01–1.47)	0.72 (0.55–0.94)	0.68 (0.38–1.20)
	Other	1.04 (0.83–1.31)	0.69 (0.50–0.97)	0.83 (0.45–1.54)
BMI category	Normal/underweight	Ref	Ref	Ref
	Overweight	1.67 (1.57–1.77)	1.82 (1.66–1.99)	1.38 (1.19–1.60)
	Obese	3.12 (2.91–3.35)	3.18 (2.85–3.55)	2.10 (1.70–2.59)
Income (per SD increase)	Continuous	0.89 (0.87–0.91)	0.84 (0.81–0.86)	0.85 (0.83–0.88)
Insurance status	Uninsured	Ref	Ref	Ref
	Insured	1.45 (1.34–1.57)	1.37 (1.20–1.58)	1.64 (1.29–2.07)
Region	Northeast	Ref	Ref	Ref
	Midwest	1.08 (1.02–1.14)	1.09 (1.00–1.19)	1.11 (1.01–1.22)
	South	1.15 (1.09–1.22)	1.13 (1.04–1.22)	1.15 (1.05–1.25)
	West	0.95 (0.89–1.01)	0.92 (0.84–1.01)	0.97 (0.87–1.09)
Interaction terms	Age × female	1.09 (1.05–1.13)***	0.92 (0.87–0.97)**	0.91 (0.83–1.00)*

OR, odds ratio; CI, confidence interval; Ref, reference category. Estimates are pooled from five multiply imputed datasets using survey-weighted logistic regression. All models adjusted for age, gender, race/ethnicity, BMI, income, insurance status, and region.

Statistical significance is denoted as follows: *p < 0.05, **p < 0.01, ***p < 0.001.

**Figure 2:**
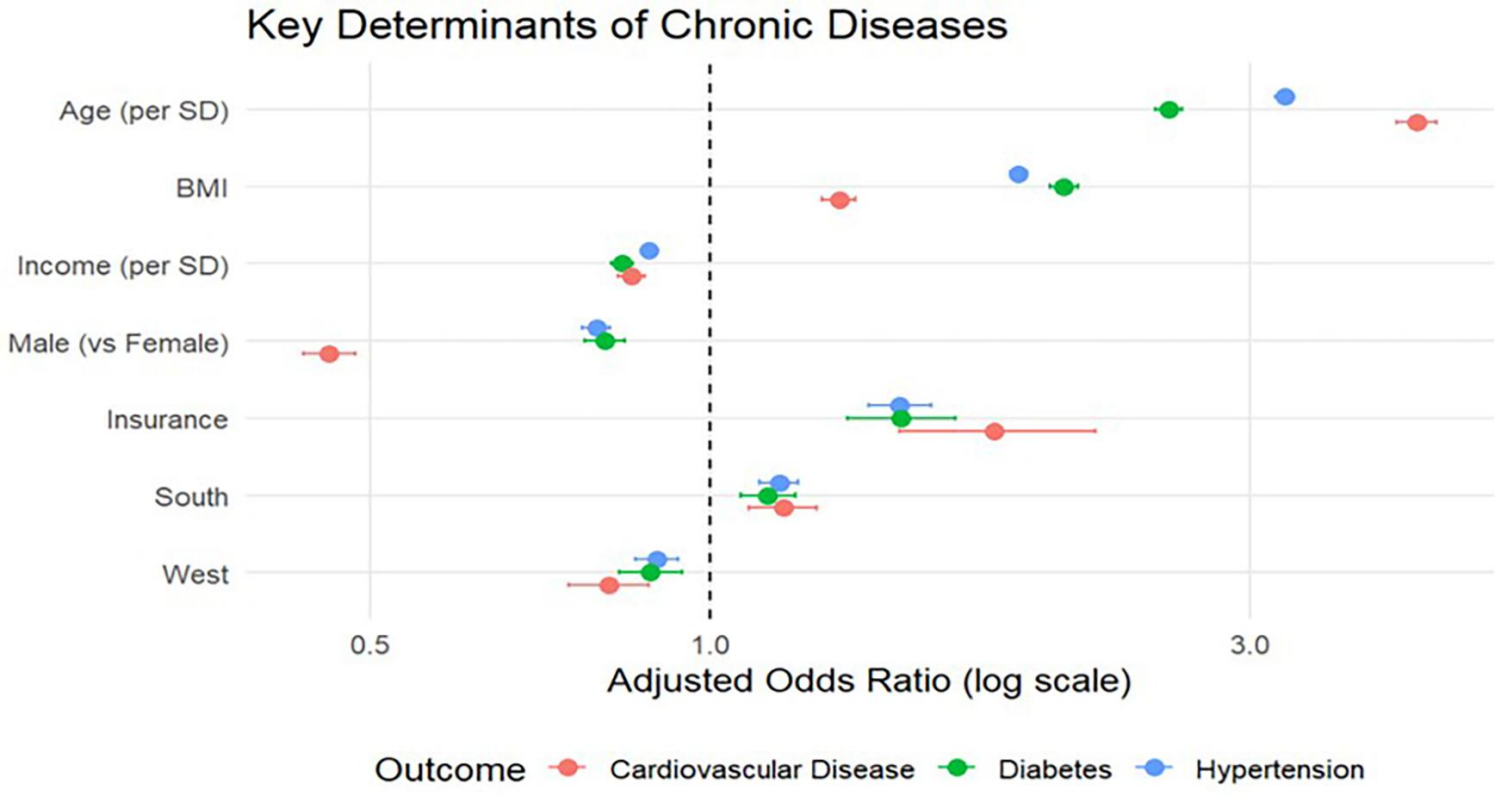
A Forest plot of adjusted odds ratios for chronic diseases.

In multivariable logistic regression analyses, age was strongly associated with all three outcomes, with each standard deviation increase corresponding to higher odds of hypertension (OR 3.30 [3.23–3.37]), diabetes (OR 2.76 [2.68–2.85]), and CVD (OR [4.08–4.52]). There were clear gender differences: females had lower odds of diabetes (OR 0.82 [0.78–0.87]), hypertension (OR 0.80 [0.77–0.83]), and CVD (OR 0.46 [0.43–0.49]) compared with males. The patterns of race and ethnicity were heterogeneous. Black American adults had higher odds of hypertension but lower odds of diabetes and CVD relative to White adults. Asian adults had slightly lower odds of hypertension but similar odds for diabetes and CVD. Other racial groups showed inconsistent associations. BMI was consistently associated with all outcomes. Obesity was associated with higher odds across all conditions (e.g., hypertension OR 3.12 [2.91–3.35]; diabetes OR 3.18 [2.85–3.55]; CVD OR 2.10 [1.70–2.59]). Higher income was associated with lower odds across all outcomes, whereas having insurance was associated with higher odds, which may influence healthcare access and diagnosis. There were modest but consistent regional differences: living in the Midwest and South was associated with slightly higher odds for all outcomes relative to the Northeast. The pattern and magnitude of these associations are visually summarized in Figure 2. These findings highlight differences in the distribution of cardiometabolic outcomes across sociodemographic groups, supporting the need for targeted interventions. Community-based interventions have demonstrated effectiveness in improving hypertension awareness, treatment, and control in similar settings (32, 33).

### 3.4 Interaction analyses

**Figure 3:**
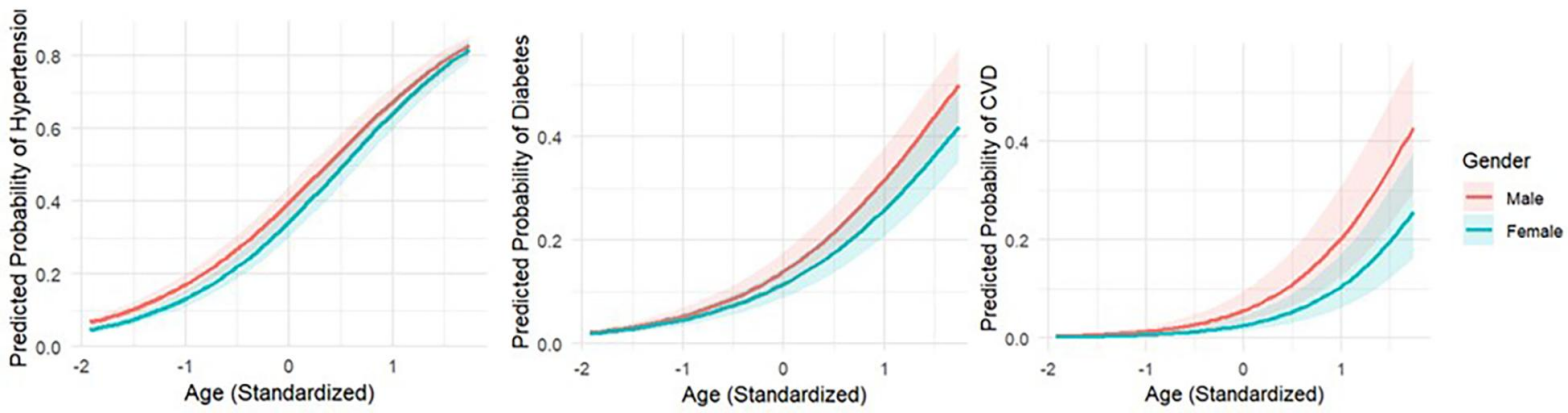
Predicted probabilities of chronic diseases across age and gender.

For every outcome, we assessed multiplicative interactions between age and gender, as well as between race/ethnicity and income; for hypertension (OR for interaction = 1.09, 95% CI: 1.05–1.13), diabetes (OR = 0.92, 95% CI: 0.87–0.97), and CVD (OR = 0.91, 95% CI: 0.83–1.00). For hypertension, the positive interaction term indicates that the age-related increase in odds was steeper among females than among males. In contrast, for diabetes and CVD, the negative interaction coefficients suggest that the age gradient was attenuated among females relative to males. These gender-differential age-related trajectories are illustrated in predicted probability plots (Figure 3). For diabetes and CVD, predicted probabilities increased more steeply with age among males, with widening gender differences at older ages. For hypertension, predicted probabilities rose steadily for both genders, with a modest but statistically significant divergence consistent with the positive interaction term. In contrast, interactions between race/ethnicity and income were not consistently statistically significant across outcomes. Although borderline effects were observed for Black American adults in relation to diabetes and CVD, confidence intervals crossed unity, and no clear pattern of effect modification was evident. These findings suggest limited evidence that the income gradient differs substantively across racial/ethnic groups within this population. Full interaction model results are provided in Supplementary Table S3. Collectively, these results indicate that gender modifies the association between age and disease outcomes, whereas income does not appear to substantially modify racial/ethnic differences in associations.

### 3.5 Model performance and calibration

**Figure 4:**
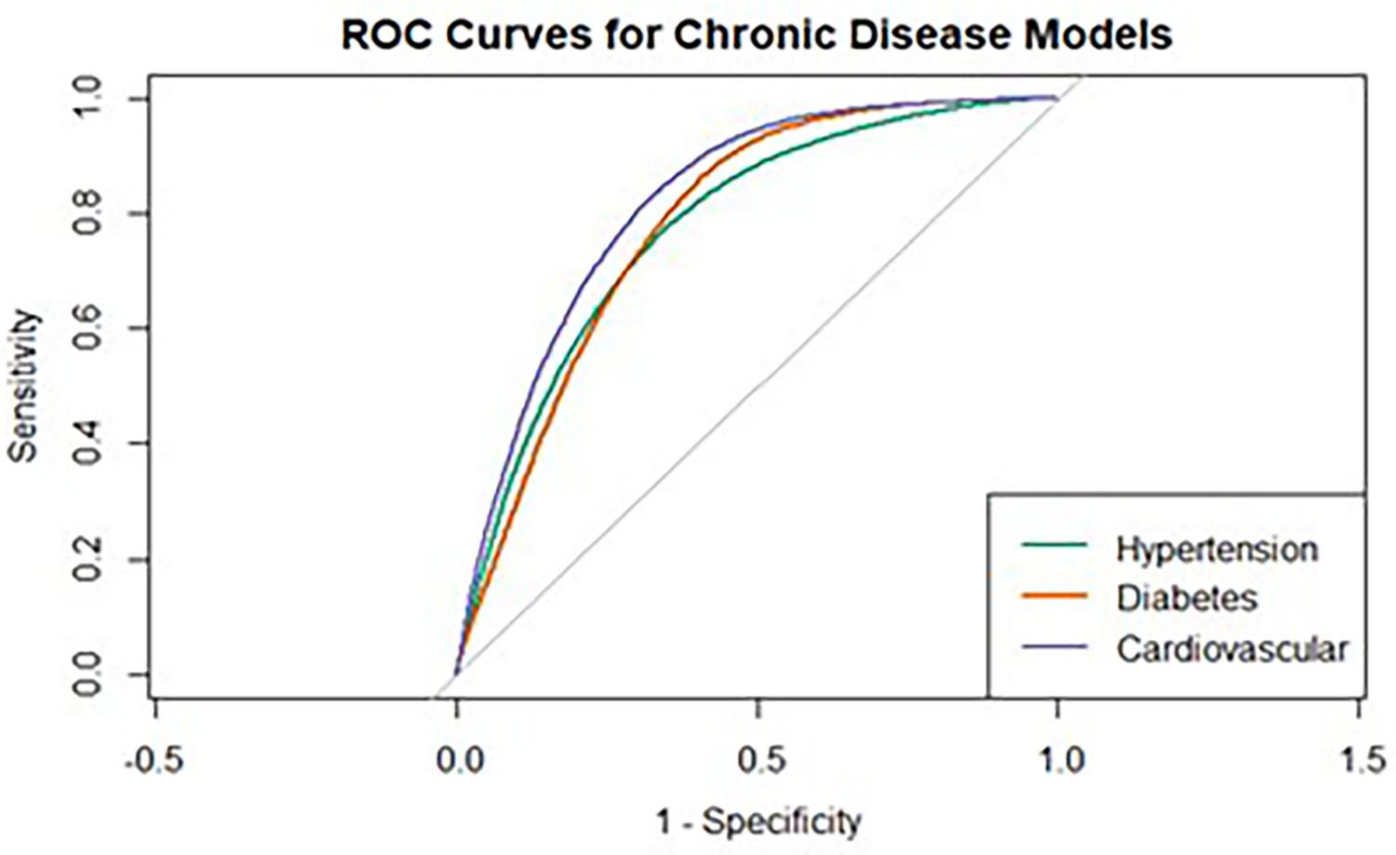
ROC curves for chronic disease models.

Model discrimination was evaluated using the area under the receiver operating characteristic curve (AUC). The hypertension model demonstrated good discriminative performance (AUC = 0.80), indicating a strong ability to differentiate between individuals with and without hypertension. The diabetes model showed acceptable discrimination (AUC = 0.77), while the CVD model exhibited the highest performance (AUC = 0.83). Together, these values indicate good overall discriminative capacity across outcomes. The ROC curves for the three models are presented in Figure 4. Model calibration was assessed using the Hosmer–Lemeshow goodness-of-fit test and graphical calibration plots. The Hosmer–Lemeshow test was statistically significant for all outcomes (*p* < 0.001), which is expected in large samples where minor deviations from perfect calibration may reach statistical significance. A visual inspection of the calibration plots (Supplementary Figures S1–S3) demonstrated good agreement between observed and predicted probabilities for all three outcomes. For hypertension, predicted probabilities closely aligned with observed values across the full range of risk. For diabetes and cardiovascular disease, minor deviations were observed at higher predicted risk levels, suggesting slight underestimation. However, overall agreement remained strong. Error bars representing 95% confidence intervals around observed estimates indicated that predicted values generally fell within the bounds of sampling variability, supporting adequate model calibration. The Hosmer–Lemeshow test results and the calibration plots are presented in Supplementary Table S2 and Supplementary Figures S1–S3, respectively.

### 3.6 Sensitivity analyses

Sensitivity analyses were performed to evaluate the robustness of the findings. Effect estimates from multiple imputation models (pooled across five imputations) were highly consistent with those from complete-case analyses, with no substantive changes in direction or magnitude of associations. This was expected given the minimal missingness in analytic variables. In addition, alternative model specifications, including variations in covariate parameterization and inclusion of interaction terms, produced results comparable to those observed in the primary models. These findings support the stability of the associations between sociodemographic and behavioral factors and the odds of hypertension, diabetes, and CVD under different analytic assumptions.

## 4 Discussion of findings

The study presents a broad view of variation in chronic conditions such as hypertension, diabetes, and cardiovascular disease (CVD) across various sociodemographic groups. In this nationally representative analysis, we identified consistent associations between sociodemographic and behavioral factors and the odds of hypertension, diabetes, and CVD. Age was strongly associated with all outcomes, with increasing age associated with substantially higher odds of chronic disease. These are consistent with those of previous studies that have also established age, gender, income, race, and region as factors associated with chronic disease (1, 34, 35). Importantly, significant age × gender interactions were observed for diabetes and CVD, indicating that age-related increases in risk were more pronounced among males. In contrast, the association between age and hypertension was similar across genders. The multivariable models demonstrated good discriminative performance (AUC range: 0.77–0.83) and adequate calibration, supporting the internal validity of the findings. Sensitivity analyses using multiple imputation and alternative model specifications yielded consistent effect estimates, reinforcing the robustness of the results. The socio-demographic disparities observed in this study align with prior population-based evidence demonstrating uneven distribution of hypertension and cardiovascular risk across populations (36, 37).

The strong association between age and chronic disease outcomes is consistent with established biological mechanisms described in prior literature, including cumulative vascular damage, metabolic dysregulation, and prolonged exposure to behavioral risk factors. However, the observed gender differences in age-related trajectories for diabetes and CVD warrant further consideration. The steeper increase in diabetes and CVD risk among males may be consistent with differences in risk factor accumulation, hormonal influences, or healthcare utilization patterns across the life course. While hypertension prevalence increased similarly with age for both genders, the more pronounced divergence in diabetes and CVD may reflect differential patterns of central adiposity, insulin resistance, or cardiometabolic risk clustering. These findings underscore the importance of considering effect modification in epidemiologic modeling. Failure to account for interactions may obscure meaningful subgroup differences and lead to oversimplified risk interpretations. Our findings align with those of the existing literature, which demonstrates higher CVD risk among males at comparable ages (38). However, the explicit modeling of age × gender interactions provides a more nuanced understanding of how risk evolves across the life course. By incorporating interaction terms into survey-weighted multivariable models, the present study extends existing literature by quantifying differential age-related trajectories rather than relying solely on stratified analyses.

Race was also significantly associated with disease outcomes. African American participants had higher odds of hypertension but lower odds of diabetes and cardiovascular disease than White subjects. This pattern is consistent with that of prior reports that proposed a disproportionately high risk of hypertension among African American populations (1). Geographically, South residents were associated with higher odds across all three disease categories, consistent with patterns reported in prior studies on the so-called “Stroke Belt” of the United States (39). Behavioural and psychosocial factors, including health beliefs and treatment adherence, may also contribute to observed disparities in chronic disease outcomes (40, 41). ROC curve analyses registered outstanding predictive capacity: the AUC was highest for the CVD model (0.82), followed by diabetes (0.80), and lowest for hypertension (approximately 0.76). Overall, these values reflect acceptable to strong discrimination, in line with established guidelines for interpreting AUC metrics (42).

From a public health perspective, the findings suggest that prevention strategies may benefit from age- and gender-tailored approaches. The sharper age-related increases in diabetes and CVD risk among males indicate a potential need for earlier or more intensive risk factor screening and intervention group. These findings are also aligned with global development priorities aimed at reducing premature mortality from non-communicable diseases (43). At the population level, the models demonstrated good discrimination, indicating their potential utility for risk stratification and identification of high-risk subgroups. However, external validation would be necessary before clinical implementation.

This study has several strengths. First, the use of nationally representative survey data enhanced generalizability. Second, survey-weighted regression modeling accounted for a complex sampling design. Third, multiple imputation was employed to address missing data, reducing potential bias associated with complete-case analysis. Finally, interaction effects were explicitly evaluated and visualized using predicted probability plots, allowing for a more interpretable assessment of effect modification.

Several limitations should be acknowledged. The cross-sectional design precludes causal inference and limits assessment of temporal relationships. Self-reported diagnoses may introduce misclassification, although such measures are commonly used in population-based research. In addition, residual confounding by unmeasured factors cannot be excluded. While model discrimination was good, predictive performance was assessed internally and may not generalize to other populations without external validation.

## 5 Summary

This study showed that sociodemographic and behavioral factors were strongly associated with hypertension, diabetes, and cardiovascular disease (CVD). Age was found to be a consistent and strong association across outcomes, with evidence of gender differences in age-related patterns for diabetes and CVD. Model performance was good, with ROC curve analyses indicating satisfactory discrimination across outcomes. These findings highlight the importance of accounting for interaction effects in epidemiological analyses and suggest that chronic disease prevention strategies may benefit from age- and gender-specific approaches. Overall, the results provide a comparative, population-level assessment of sociodemographic gradients in chronic disease and establish a basis for future longitudinal investigation.

## Data Availability

Publicly available datasets were analyzed in this study. These datasets can be found here: https://nhis.ipums.org/nhis-action/variables/group.
