# Supplementary material for: An epidemiological assessment of the distribution and sociodemographic burden of chronic diseases: a focus on hypertension, diabetes, and cardiovascular conditions
Source: Front Epidemiol. 2026 May 22;6:1715893. doi: 10.3389/fepid.2026.1715893 (PMC13236920; doi:10.3389/fepid.2026.1715893)
Supplement: Supplementary file 1 [file Table1.docx]

**Supplementary**

Table S1: Age-stratified prevalence of hypertension by gender

| Age group | Male prevalence | Female prevalence |
| --- | --- | --- |
| 18-39 | 9.9% (SE 0.0030) | 8.2% (SE 0.0028) |
| 40-59 | 32.3% (SE 0.0047) | 26.4% (SE 0.0043) |
| 60-79 | 58.2% (SE 0.0047) | 52.4% (SE 0.0044) |
| 80+ | 65.8% (SE 0.0086) | 68.6% (SE 0.0076) |

Table S2: Hosmer and Lemeshow Goodness of fit test for Logistic Regression Models

| Outcome | Chi-square (χ²) | df | p-value |
| --- | --- | --- | --- |
| Hypertension | 324.86 | 8 | <0.001*** |
| Diabetes | 50,422.00 | 8 | <0.001*** |
| Cardiovascular Disease | 65,913.00 | 8 | <0.001*** |

Table S3: Interaction Effects for Hypertension, Diabetes, and Cardiovascular Disease

| Interaction term | Hypertension OR (95% CI) | p-value | Diabetes OR (95% CI) | p-value | CVD OR (95% CI) | p-value |
| --- | --- | --- | --- | --- | --- | --- |
| Age × Female | 1.09 (1.05–1.13) | <0.001 | 0.92 (0.87–0.97) | 0.001 | 0.91 (0.83–1.00) | 0.046 |
| Asian × Income | 1.10 (0.89–1.35) | 0.387 | 1.15 (0.94–1.40) | 0.175 | 0.85 (0.61–1.20) | 0.342 |
| African American × Income | 1.05 (0.86–1.28) | 0.629 | 1.19 (1.00–1.42) | 0.054 | 0.76 (0.56–1.03) | 0.077 |
| Other × Income | 0.98 (0.77–1.23) | 0.843 | 0.89 (0.68–1.16) | 0.396 | 0.90 (0.60–1.36) | 0.608 |
| Unknown/Refused × Income | 1.07 (0.87–1.32) | 0.529 | 1.08 (0.89–1.30) | 0.406 | 0.79 (0.56–1.12) | 0.185 |
| White × Income | 1.05 (0.87–1.27) | 0.587 | 1.07 (0.91–1.26) | 0.435 | 0.82 (0.61–1.10) | 0.185 |


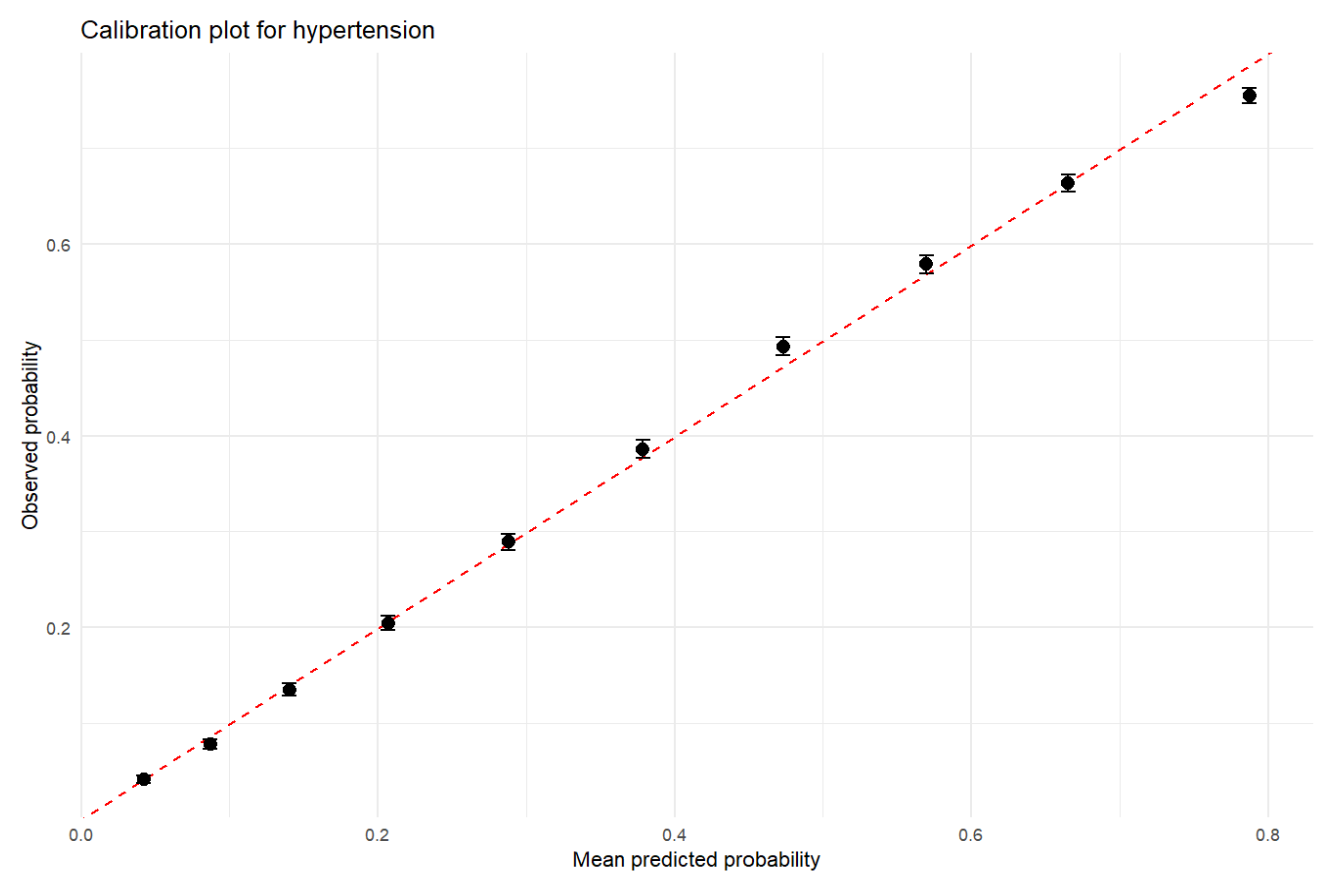


Fig S1: Calibration plot of predicted probabilities for hypertension


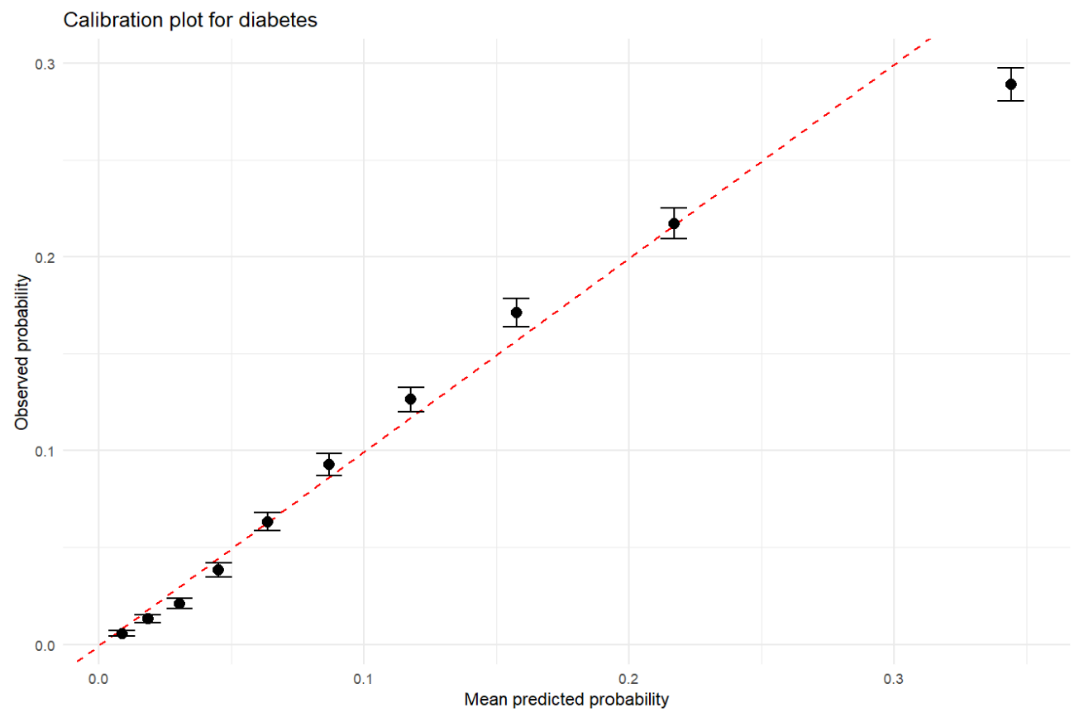


Fig S2: Calibration plot of predicted probabilities for diabetes


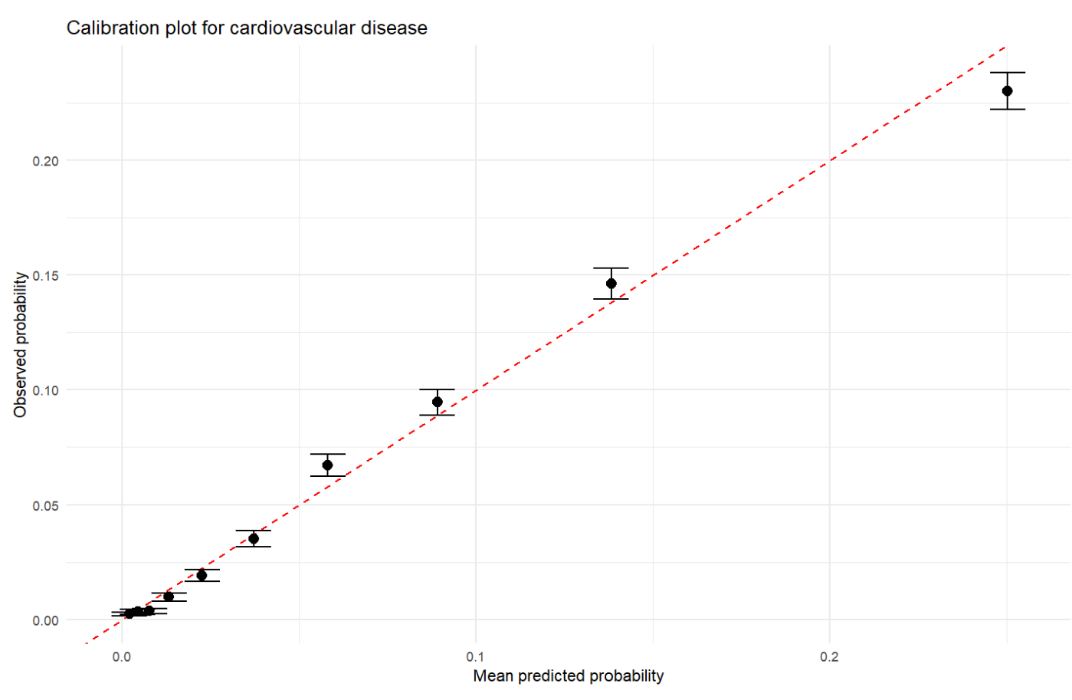


Fig S3: Calibration plot of predicted probabilities for cardiovascular disea


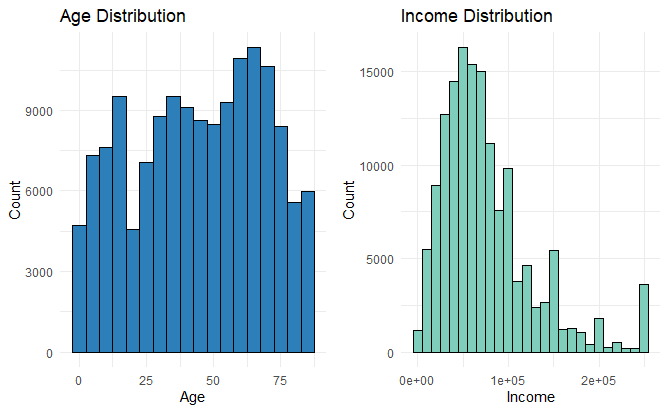


Fig S4 showing the Age and income distribution of respondents.


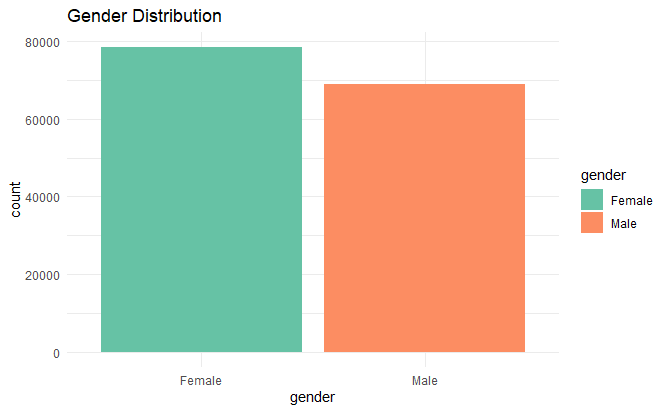


Fig S5 showing the Gender distribution of respondents.


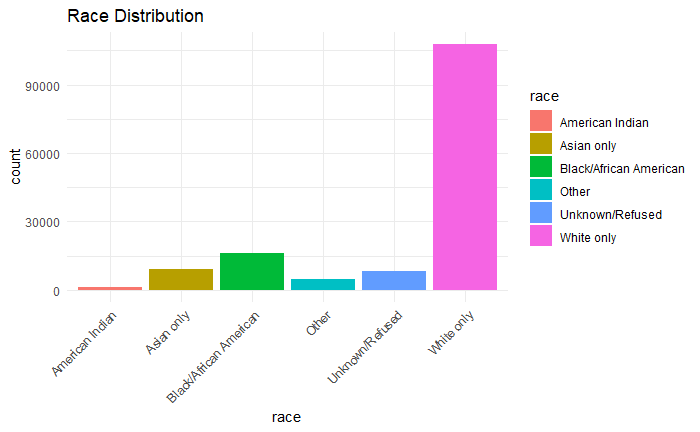


Fig S6 showing the various race of respondents.


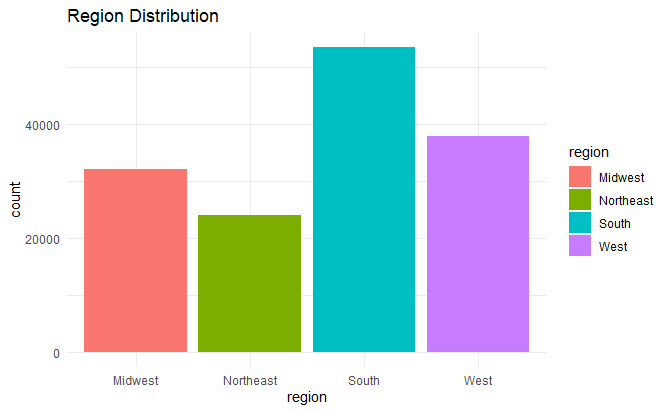


Fig S7 showing the distribution of Regions of respondents.


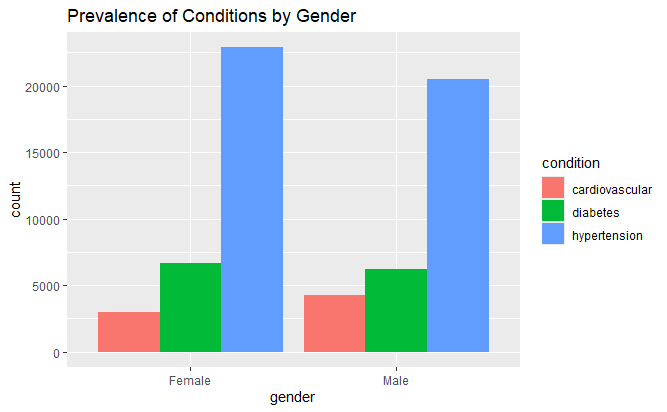


Fig S8 showing the prevalence condition by gender


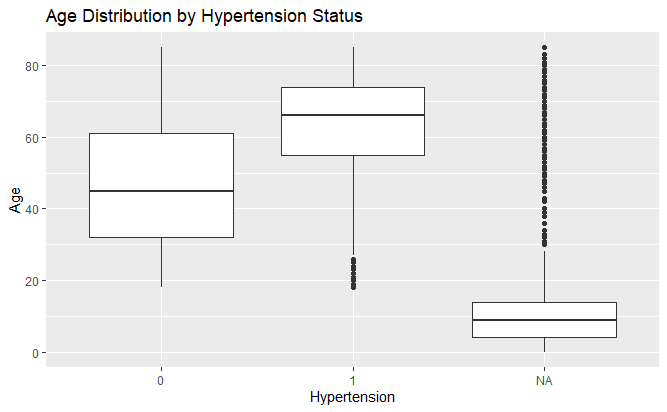


Fig S9 Box plot of age distribution by hypertension


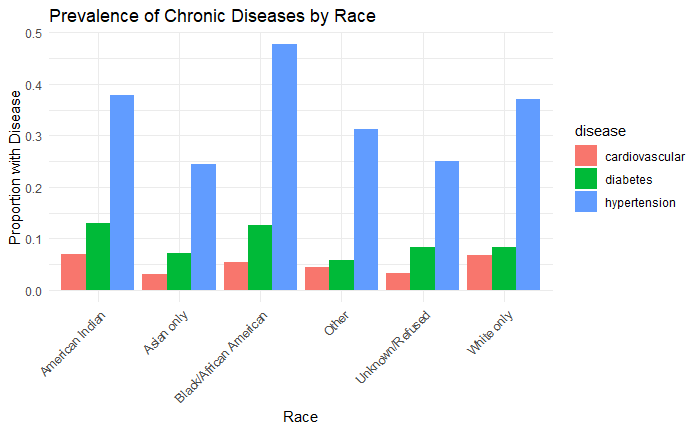


Fig S10 showing the prevalence of chronic diseases by race


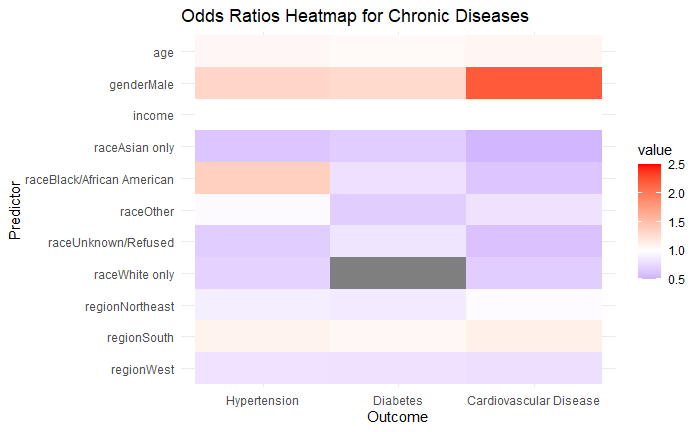


Fig S11 A heatmap showing the odds ratios for chronic diseases.


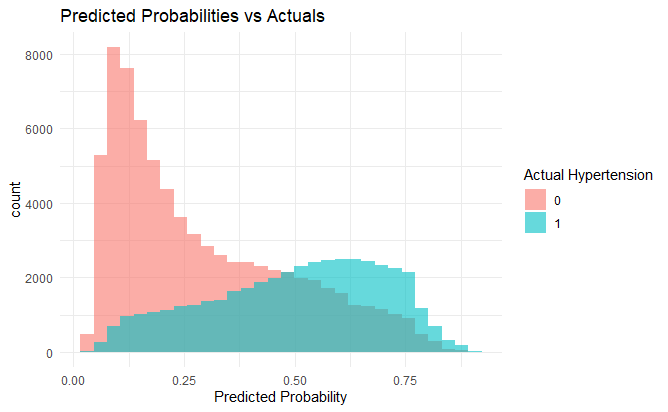


Fig S12 Predicted probabilities against the actual

**Crude prevalence table**

|  | **age** | **HTN** | **DM** | **CVD** | **HTN_se** | **DM_se** | **CVD_se** | **determinant** |
| --- | --- | --- | --- | --- | --- | --- | --- | --- |
|  |  |  |  |  |  |  |  |  |
| **18** | **18** | **2.047569** | **0.3616751** | **0.00000000** | **0.005037594** | **0.002099610** | **0.0000000000** | **Age** |
| **19** | **19** | **4.866259** | **0.5069349** | **0.90383672** | **0.008849165** | **0.002421294** | **0.0039176646** | **Age** |
| **20** | **20** | **5.001627** | **0.5464693** | **0.26940003** | **0.009822691** | **0.002657733** | **0.0019762729** | **Age** |
| **21** | **21** | **4.702418** | **1.1943916** | **0.38555310** | **0.008856295** | **0.005568941** | **0.0023145582** | **Age** |
| **22** | **22** | **4.517275** | **1.3344795** | **0.10908226** | **0.007326492** | **0.004034922** | **0.0008859470** | **Age** |
| **23** | **23** | **5.952966** | **1.2123381** | **0.15109316** | **0.009764567** | **0.004504656** | **0.0011846568** | **Age** |
| **24** | **24** | **4.825242** | **1.1491967** | **0.76327185** | **0.006881656** | **0.004219975** | **0.0039953249** | **Age** |
| **25** | **25** | **7.002370** | **0.7625839** | **0.17121743** | **0.008242994** | **0.002500292** | **0.0012563586** | **Age** |
| **26** | **26** | **8.918016** | **1.5227033** | **0.37379217** | **0.009617908** | **0.004383654** | **0.0015421042** | **Age** |
| **27** | **27** | **7.723277** | **1.5520964** | **0.61131129** | **0.009431690** | **0.003986540** | **0.0034964806** | **Age** |
| **28** | **28** | **9.700326** | **1.0207209** | **0.08935134** | **0.009302218** | **0.002845352** | **0.0008942775** | **Age** |
| **29** | **29** | **8.058011** | **0.6955879** | **0.28495671** | **0.007869870** | **0.002155529** | **0.0013848369** | **Age** |
| **30** | **30** | **9.500808** | **1.7933340** | **0.26931190** | **0.009233502** | **0.003916327** | **0.0013077657** | **Age** |
| **31** | **31** | **10.995139** | **1.0546938** | **0.19985052** | **0.009174113** | **0.002817949** | **0.0011983627** | **Age** |
| **32** | **32** | **11.138840** | **1.2865319** | **0.29003203** | **0.009048183** | **0.003525786** | **0.0014497136** | **Age** |
| **33** | **33** | **10.799763** | **2.4164487** | **0.19110759** | **0.009017715** | **0.004628668** | **0.0019093344** | **Age** |
| **34** | **34** | **12.838172** | **2.3067344** | **0.42613012** | **0.009379014** | **0.004032821** | **0.0020096099** | **Age** |
| **35** | **35** | **13.826933** | **1.3761739** | **0.04713909** | **0.009445884** | **0.003158723** | **0.0004714187** | **Age** |
| **36** | **36** | **13.535738** | **2.7948917** | **0.48259504** | **0.009275398** | **0.004205584** | **0.0020699586** | **Age** |
| **37** | **37** | **15.803618** | **3.3733325** | **0.68897067** | **0.010659057** | **0.005294374** | **0.0021800423** | **Age** |
| **38** | **38** | **15.864311** | **3.4500936** | **0.42530550** | **0.011204164** | **0.005215760** | **0.0017658108** | **Age** |
| **39** | **39** | **16.488324** | **3.5527142** | **0.62321395** | **0.010831369** | **0.005981924** | **0.0022413528** | **Age** |
| **40** | **40** | **17.670723** | **4.3327982** | **0.66008690** | **0.010706981** | **0.005753687** | **0.0021914436** | **Age** |
| **41** | **41** | **19.416417** | **4.0832192** | **0.61730596** | **0.010866526** | **0.006296365** | **0.0027290829** | **Age** |
| **42** | **42** | **20.069135** | **4.3618837** | **0.65697158** | **0.012153630** | **0.006352811** | **0.0023240541** | **Age** |
| **43** | **43** | **21.269232** | **5.4058298** | **0.77763937** | **0.011274110** | **0.006264474** | **0.0026996762** | **Age** |
| **44** | **44** | **22.852262** | **5.2844157** | **1.22415756** | **0.012624162** | **0.006338262** | **0.0038116979** | **Age** |
| **45** | **45** | **25.708777** | **6.2969582** | **1.11706403** | **0.013497718** | **0.007478323** | **0.0036915816** | **Age** |
| **46** | **46** | **25.787801** | **6.6755379** | **1.21759149** | **0.011866289** | **0.007822446** | **0.0038234629** | **Age** |
| **47** | **47** | **27.873961** | **8.4878114** | **2.33371298** | **0.014208331** | **0.008631154** | **0.0053732146** | **Age** |
| **48** | **48** | **27.204639** | **7.3717672** | **1.39695918** | **0.013488634** | **0.008146006** | **0.0042336400** | **Age** |
| **49** | **49** | **31.715526** | **10.6115571** | **2.55917675** | **0.013780276** | **0.009780093** | **0.0052827210** | **Age** |
| **50** | **50** | **30.612195** | **8.1450620** | **3.19490998** | **0.014201975** | **0.008586603** | **0.0048754628** | **Age** |
| **51** | **51** | **33.738573** | **9.4711270** | **2.12375450** | **0.015416964** | **0.008666548** | **0.0039716807** | **Age** |
| **52** | **52** | **34.394924** | **11.2698007** | **2.84008828** | **0.014028121** | **0.009437944** | **0.0050754942** | **Age** |
| **53** | **53** | **34.799366** | **10.1703286** | **3.73896344** | **0.013937718** | **0.008700090** | **0.0054983803** | **Age** |
| **54** | **54** | **35.716224** | **11.4283841** | **3.19232003** | **0.013885741** | **0.008853435** | **0.0048054076** | **Age** |
| **55** | **55** | **37.787905** | **13.3421486** | **3.86017134** | **0.013975306** | **0.011103025** | **0.0056212587** | **Age** |
| **56** | **56** | **41.792008** | **12.2766602** | **4.76087681** | **0.013750555** | **0.009259687** | **0.0060312616** | **Age** |
| **57** | **57** | **40.597432** | **12.3978541** | **4.93891623** | **0.012574505** | **0.009263802** | **0.0064646110** | **Age** |
| **58** | **58** | **41.512914** | **13.0259761** | **4.34183395** | **0.013125855** | **0.009361769** | **0.0049149979** | **Age** |
| **59** | **59** | **43.098457** | **12.5688963** | **6.25481585** | **0.013094146** | **0.008855179** | **0.0069107825** | **Age** |
| **60** | **60** | **46.776562** | **15.3211051** | **6.07790087** | **0.013201866** | **0.009959522** | **0.0063129033** | **Age** |
| **61** | **61** | **46.985812** | **14.3198504** | **6.56882361** | **0.013536696** | **0.009042276** | **0.0067188494** | **Age** |
| **62** | **62** | **51.174488** | **15.6084895** | **6.64218712** | **0.012848241** | **0.009369924** | **0.0064518677** | **Age** |
| **63** | **63** | **52.034257** | **15.3972952** | **7.20653524** | **0.012919231** | **0.009377796** | **0.0066766588** | **Age** |
| **64** | **64** | **50.630127** | **18.0400472** | **7.77950191** | **0.013091234** | **0.010543708** | **0.0069223683** | **Age** |
| **65** | **65** | **51.486128** | **17.1380352** | **9.16837623** | **0.013027647** | **0.010357585** | **0.0069334863** | **Age** |
| **66** | **66** | **53.029807** | **18.3233098** | **8.90572698** | **0.012896803** | **0.009945203** | **0.0070472621** | **Age** |
| **67** | **67** | **53.184470** | **19.1243736** | **10.28997223** | **0.013242953** | **0.011328964** | **0.0082537469** | **Age** |
| **68** | **68** | **54.622395** | **18.5098247** | **10.57244419** | **0.012724291** | **0.010274931** | **0.0079868355** | **Age** |
| **69** | **69** | **56.729036** | **19.3315045** | **10.84351993** | **0.013564689** | **0.010304043** | **0.0082270661** | **Age** |
| **70** | **70** | **59.068040** | **19.8456968** | **11.87841744** | **0.012599538** | **0.011038705** | **0.0090461432** | **Age** |
| **71** | **71** | **60.159588** | **20.3634025** | **11.54131854** | **0.013308330** | **0.011976826** | **0.0087086733** | **Age** |
| **72** | **72** | **61.531351** | **20.5425965** | **12.54469473** | **0.013381095** | **0.011024012** | **0.0089525398** | **Age** |
| **73** | **73** | **65.114672** | **21.8542314** | **13.62619068** | **0.013465636** | **0.010975995** | **0.0089600840** | **Age** |
| **74** | **74** | **66.263368** | **22.5132180** | **15.01685146** | **0.013752453** | **0.011989140** | **0.0102888674** | **Age** |
| **75** | **75** | **64.616803** | **21.3042304** | **16.52025071** | **0.014206255** | **0.013075732** | **0.0105927901** | **Age** |
| **76** | **76** | **65.539547** | **20.4079596** | **15.26439011** | **0.015104959** | **0.011937936** | **0.0106196370** | **Age** |
| **77** | **77** | **66.039417** | **24.1854625** | **17.69257897** | **0.015267799** | **0.014341821** | **0.0125769747** | **Age** |
| **78** | **78** | **66.139773** | **20.3461345** | **19.20219691** | **0.015696821** | **0.014588546** | **0.0134217584** | **Age** |
| **79** | **79** | **65.834159** | **19.5071944** | **20.82093662** | **0.016529471** | **0.013766012** | **0.0132423952** | **Age** |
| **80** | **80** | **68.164177** | **18.1924916** | **18.40311114** | **0.017639478** | **0.015318667** | **0.0145986974** | **Age** |
| **81** | **81** | **70.115643** | **23.1868091** | **21.29047122** | **0.017826651** | **0.016303029** | **0.0174091580** | **Age** |
| **82** | **82** | **64.262294** | **23.9506593** | **20.13658791** | **0.019052968** | **0.018947206** | **0.0160213408** | **Age** |
| **83** | **83** | **66.015385** | **20.5004939** | **21.19359139** | **0.021117500** | **0.017820592** | **0.0171208862** | **Age** |
| **84** | **84** | **70.007151** | **19.5788129** | **20.83204387** | **0.020491990** | **0.017100306** | **0.0175136575** | **Age** |
| **85** | **85** | **67.536659** | **16.9812386** | **22.99123088** | **0.009078499** | **0.007802640** | **0.0082811894** | **Age** |
| **1...69** | ***NA*** | **32.751614** | **9.7495400** | **6.22125496** | **0.002875128** | **0.001688091** | **0.0013379857** | **Sex** |
| **2...70** | ***NA*** | **29.759368** | **8.6938701** | **3.48870670** | **0.002825947** | **0.001614516** | **0.0008978682** | **Sex** |
| **American Indian** | ***NA*** | **31.528807** | **13.7161081** | **4.64271676** | **0.018539645** | **0.015665354** | **0.0133878560** | **Race** |
| **Asian only** | ***NA*** | **23.895980** | **9.9769839** | **2.93329846** | **0.007488268** | **0.004814542** | **0.0027185049** | **Race** |
| **Black/African American** | ***NA*** | **39.249153** | **12.4357610** | **4.21361376** | **0.006307015** | **0.003873153** | **0.0022929175** | **Race** |
| **Other** | ***NA*** | **26.638399** | **8.1208531** | **3.10398097** | **0.012045866** | **0.006986789** | **0.0036979703** | **Race** |
| **Unknown/Refused** | ***NA*** | **22.826199** | **10.5834326** | **2.62986751** | **0.007441715** | **0.005243812** | **0.0023780070** | **Race** |
| **White only** | ***NA*** | **31.406097** | **8.4702365** | **5.33341745** | **0.002549898** | **0.001335881** | **0.0009763424** | **Race** |
| **1...77** | ***NA*** | **16.280408** | **2.9414482** | **2.58250606** | **0.013137658** | **0.007134046** | **0.0045660939** | **BMI** |
| **2...78** | ***NA*** | **19.688323** | **4.5061447** | **3.75437348** | **0.002850296** | **0.001383155** | **0.0012184386** | **BMI** |
| **3** | ***NA*** | **31.662715** | **8.7399556** | **5.08290202** | **0.003060810** | **0.001815945** | **0.0013207933** | **BMI** |
| **4** | ***NA*** | **42.939630** | **14.7287762** | **5.68911545** | **0.003414578** | **0.002459257** | **0.0014164142** | **BMI** |
| **0** | ***NA*** | **34.266548** | **12.4499318** | **6.02514356** | **0.005868143** | **0.003646868** | **0.0023383761** | **Income** |
| **0.5** | ***NA*** | **31.399695** | **9.1902407** | **4.80801117** | **0.002380135** | **0.001312052** | **0.0009218795** | **Income** |
| **1...83** | ***NA*** | **26.875008** | **5.9231248** | **3.65830820** | **0.004697659** | **0.002258808** | **0.0017459741** | **Income** |
| **Midwest** | ***NA*** | **31.126637** | **8.8547762** | **4.96417323** | **0.004851237** | **0.002670782** | **0.0018070465** | **Region** |
| **Northeast** | ***NA*** | **30.534372** | **8.6933174** | **5.02792516** | **0.005135333** | **0.002807345** | **0.0020202790** | **Region** |
| **South** | ***NA*** | **34.164130** | **10.2879860** | **5.41765516** | **0.003631675** | **0.002138663** | **0.0014199731** | **Region** |
| **West** | ***NA*** | **27.092378** | **8.1728213** | **3.59666569** | **0.004790669** | **0.002566332** | **0.0015465700** | **Region** |
